# Multiple Levels of Influence on Lifestyle Behaviors among Cancer Survivors in Racial and Ethnic Minority Groups: A Systematic Review

**DOI:** 10.1155/2023/8504968

**Published:** 2023-07-13

**Authors:** Dalnim Cho, Seokhun Kim, Scherezade K. Mama, Maria C. Swartz, Yimin Geng, Qian Lu

**Affiliations:** 1 Department of Health Disparities Research, The University of Texas MD Anderson Cancer Center, Houston, TX, USA; 2 Center for Clinical Research and Evidence-Based Medicine, The University of Texas McGovern Medical School, Houston, TX, USA; 3 Department of Pediatrics-Research, The University of Texas MD Anderson Cancer Center, Houston, TX, USA; 4 Research Medical Library, The University of Texas MD Anderson Cancer Center, Houston, TX, USA

## Abstract

**Objective.:**

This systematic review aimed to provide a critical summary of studies of physical activity (PA) and diet among racial/ethnic minority cancer survivors. Guided by the socio-ecological model, we identified factors across multiple levels—individual, family/social support, provider/team, and organization/local community/policy environment—that affect PA and diet among racial/ethnic minority survivors.

**Methods.:**

We searched the Ovid MEDLINE, EBSCO CINAHL, Ovid PsycInfo, and PubMed databases. We extracted the behavior of focus (i.e., PA and diet), cancer type, race/ethnicity, and the level(s) of influence (and the corresponding factor(s)), and each eligible study investigated individual (e.g., demographic characteristics, psychological factors), family/social support, provider/team (e.g., healthcare provider recommendations), and organization/local community/policy environment (e.g., neighborhood/social environment).

**Results.:**

Of 1,603 studies identified, 23 unique studies were eligible. Most studies included breast cancer survivors (*n* = 19) and Black survivors (*n* = 13). Seventeen studies assessed associations between PA and factors at the level of the individual (16 studies), family/social support (two studies), provider/team (one study), or organization/local community/policy environment (four studies). Eleven studies assessed associations between diet and factors at the level of the individual (11 studies), family/social support (two studies), provider/team (one study), or organization/local community/policy environment (two studies). Only five studies simultaneously investigated factors across multiple levels. Most demographic and cancer-related factors were not associated with PA or diet. Overall, factors from social-cognitive theories (e.g., self-efficacy) were positively associated with PA. Less consensus was found regarding diet because fewer studies existed, and they also investigated a diverse range of eating behaviors.

**Conclusions.:**

There is a critical need for studies of PA and diet that investigate multiple levels of influence particularly for Asian American survivors, male survivors, and cancers other than breast cancer. Social-cognitive theories may help guide the designing of multilevel PA interventions for racial/ethnic minority survivors. Studies assessing overall eating quality or adherence to dietary guidelines are needed.

## 1. Background

Racial/ethnic minority populations, including Black, Hispanic/Latino, Asian and Pacific Islander, and American Indian and Alaska Native populations, are increasing rapidly in the United States [1] and thus changing the demographics of the country’s cancer patient population. Between 2010 and 2030, the cancer incidence in the United States is expected to increase by 45% [2], and this increase is driven in part by the growth of racial/ethnic minority populations. Whereas cancer incidence is expected to increase 31% among non-Hispanic White (NHW) adults, it is expected to increase 99% among all racial/ethnic minority adults, with increases of 64% for Black adults, 132% for Asian/Pacific Islander adults, and 142% for Hispanic adults [2]. Thus, efforts to improve the health of racial/ethnic minority cancer survivors are more important than ever.

Healthy lifestyle behaviors, particularly physical activity (PA) and healthy eating (e.g., consuming fruits and vegetables, limiting the consumption of red or processed meats, abstaining from alcohol), are critical for cancer survivors’ health. PA and healthy eating can reduce cancer-specific and all-cause mortality among cancer survivors [3–6], and PA in particular increases cancer survivors’ quality of life (QoL) [7, 8]. However, racial/ethnic minority cancer survivors’ lifestyle behaviors are not optimal. Compared with NHW survivors, Black survivors are more likely to be physically inactive, not adhere to fruit and vegetable intake guidelines, and have overweight or obesity [9, 10], and Hispanic survivors are more likely to not adhere to fruit and vegetable intake guidelines and have overweight or obesity [10]. Asian cancer survivors are less likely than NHW survivors to have overweight or obesity, but they are more likely to not meet PA guidelines [9].

Understanding and ultimately improving racial/ethnic minority survivors’ PA and eating behaviors requires a comprehensive investigation of the factors that influence those behaviors. A socio-ecological approach, which posits that lifestyle behaviors are shaped by multiple levels of influence, such as intrapersonal, social/cultural, natural, and policy environments, can help elucidate those levels and identify factors across multiple levels that influence PA and eating behaviors [11, 12]. In the context of cancer survivorship, in addition to individual characteristics (e.g., sociodemographic characteristics and psychological factors such as knowledge, attitudes, and beliefs), many other factors influence cancer care quality, which in turn impacts cancer-related health outcomes [13]. These other factors include elements of family/social support (e.g., family dynamics, friends, network support), the healthcare provider/team (e.g., provider’s/team’s knowledge, communication skills, cultural competency), the organization/practice setting (e.g., organizational structure, patient education, and navigation), the local community environment (e.g., lay support networks in local community, market structure in local hospital and cancer services), state health policy (e.g., Medicaid reimbursement, state cancer plans/programs), and national health policy (e.g., national cancer initiatives, professional standards). Investigating these multiple levels of influence is critical to understanding racial/ethnic minority cancer survivors’ lifestyle behaviors because these survivors are more likely than NHW survivors to experience barriers to access to care, including financial, transportation, and organizational barriers [14], that may not be effectively resolved by targeting only individual-level factors such as knowledge or willpower.

Many interventions to improve PA and eating behaviors among cancer survivors have been developed, but most of these interventions were developed and tested with study populations of predominantly NHW cancer survivors, and PA and diet interventions for racial/ethnic minority populations remain scarce [15, 16]. Thus, whether such interventions can successfully improve racial/ethnic minority survivors’ PA and eating behaviors is unclear. In addition, despite the importance of addressing multiple levels of influence for minority survivors, translating this knowledge into the development of a multilevel lifestyle intervention can be challenging, given that there is no consensus on which factors at each level of influence need to be targeted. In fact, the field of multilevel intervention research lacks a single unifying theory [17] to guide the selection of levels and their corresponding factors. Therefore, studies that synthesize existing empirical knowledge are urgently needed to inform the development of effective multilevel interventions to improve racial/ethnic minority cancer survivors’ PA and eating behaviors.

Accordingly, guided by the socio-ecological framework for cancer survivorship [13], we conducted a systematic review to identify the levels of influence and their corresponding factors that affect the PA and eating behaviors of racial/ethnic minority cancer survivors. This systematic review study is reported according to the updated Preferred Reporting Items for Systematic Reviews and Meta-Analyses (PRISMA) guidelines [18].

## 2. Methods

### 2.1. Search Strategies.

We searched electronic databases, including Ovid MEDLINE (established in 1946), EBSCO CINAHL (1986), Ovid PsycInfo (1967), and PubMed, for articles published from database inception to June 7, 2021. We also searched PubMed on June 7, 2021, for articles published online ahead of print. The following subject headings and keywords were used in the search: “cancer,” “neoplasms,” “tumor,” “leukemia,” “lymphoma,” “carcinoma,” “malignancy,” “oncology,” “survivors,” “patients,” “population,” “ethnic groups,” “racial groups,” “minority groups,” “African Americans,” “Asian Americans,” “Hispanic Americans,” “health behavior,” “lifestyle,” “exercise,” “alcohol drinking,” “diet,” and “activities of daily living.” Search terms were combined by “or” if they represented similar concepts or were combined by “and” if they represented different concepts. Search structures, subject headings, and keywords were tailored to each database. A complete list of the search strategies is provided in the supplementary material (Tables S1–S4). Abstract reviews and full article reviews were conducted by the second author (SK) and the first author (DC), respectively.

### 2.2. Inclusion/Exclusion Criteria.

Eligible studies examined associations between the aforementioned factors (e.g., psychological factors, family/social support, local community environment) and PA and/or eating behaviors (defined as specific eating behaviors (e.g., fruit and vegetable intake, red meat/processed meat intake, sugary drink consumption, alcohol use) or overall eating quality) and included cancer survivors at least 18 years old. Studies that included multiple race/ethnicity groups were eligible if their populations were at least 50% racial/ethnic minority cancer survivors or if they provided a subgroup analysis by race/ethnicity. Studies were not excluded based on cancer type or stage. Experimental/interventional studies were included if they had results about associations between factors and lifestyle behaviors. Qualitative studies and book chapters that did not explicitly examine associations between factors and lifestyle behaviors were excluded. Studies other than full research articles (e.g., commentaries, research protocols, conference abstracts, dissertations), studies published in languages other than English, and studies conducted outside the United States were excluded.

### 2.3. Coding.

For each eligible study, we used Microsoft Excel to record the sample size and patient characteristics (size, race/ethnicity, gender, age, cancer type, cancer stage, cancer treatments, time since diagnosis), study design (e.g., cross-sectional, longitudinal), target behaviors (e.g., PA and/or eating behaviors), and factors across levels of influence. We considered four levels of influence and their corresponding factors based on the socio-ecological framework [13]: (1) individual (e.g., demographics, psychological factors), (2) family/social (e.g., support from family and friends), (3) provider/care team (e.g., physician knowledge, communication skills, cultural competency), and (4) organization/local community/policy environment (e.g., neighborhood, built environment, social environment, acculturation). The last level was a combination of multiple levels of influence because very few studies examined these levels of influence. If a study measured factors and lifestyle behaviors multiple times and thus reported multiple associations, we selected and reported longitudinal ones (e.g., associations between a factor at time 1 and a lifestyle behavior at time 2). The quality of individual studies was evaluated using the Critical Appraisal Skills Program (CASP) checklist [19] for randomized controlled trials and the adapted Appraisal tool for Cross-Sectional Studies (AXIS tool) [20] for observational studies. Because both the CASP checklist and AXIS tool do not provide a scored list of items that summarize judgments of quality (e.g., good, fair, and poor), for each study, we divided the number of criteria met by the total number of criteria included on the CASP checklist or AXIS tool to obtain a proportion describing the quality of the study. The lead author (DC) conducted the coding and quality check.

## 3. Results

**Figure 1:**
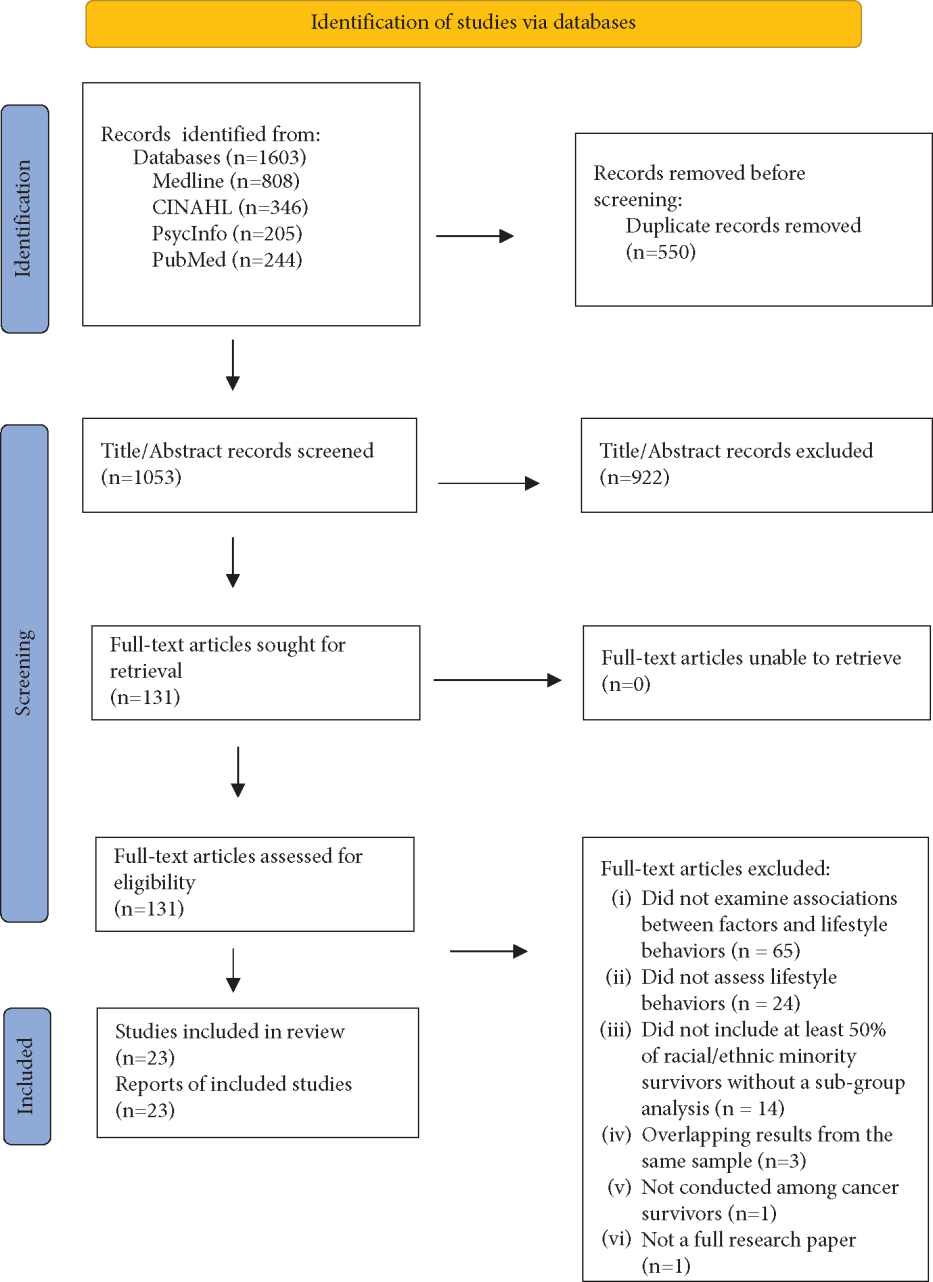
Flowchart of the literature search.

The literature search flowchart is shown in Figure 1. The electronic database search retrieved 1,603 records. After removing duplicate records (*n* = 550), we identified 1,053 unique records. Of those, 922 were excluded during the abstract review because they did not assess lifestyle behaviors (*n* = 463), did not include cancer survivors (*n* = 228), did not examine the association between factors and lifestyle behaviors (*n* = 133), were not full research papers (*n* = 60), were not conducted in the United States (*n* = 19), did not include racial/ethnic minority cancer survivors (*n* = 13), or did not have a population that was at least 50% racial/ethnic minority survivors without a subgroup analysis (*n* = 6).

Of the remaining 131 articles included in the full-text review, 108 were excluded because they did not examine associations between factors and lifestyle behaviors (*n* = 65), did not assess lifestyle behaviors (*n* = 24), did not have a population that was at least 50% racial/ethnic minority survivors without a subgroup analysis (*n* = 14), reported overlapping results from the same sample(s) (*n* = 3), did not include cancer survivors (*n* = 1), or were not full research papers (*n* = 1). Thus, a total of 23 unique studies comprising a total of 5,959 participants were included in this review.

### 3.1. Study Characteristics.

The characteristics of the 23 studies included in this review are given in Table 1 (see Table S5 in the supplementary material as well). Of these studies, 57% included Black cancer survivors (*n* = 13 studies of 6 populations), and 83% included female breast cancer survivors (*n* = 19 studies of 12 populations). Two studies were randomized controlled trials, and 21 studies were observational studies. Of the 23 studies, 16 (70%) were cross-sectional studies (or cross-sectional analyses of longitudinal or interventional studies), and seven (30%) were longitudinal studies. Twelve studies (52%) investigated PA only, six (26%) investigated eating behaviors only, and five (22%) investigated both PA and eating behaviors. All 17 studies of PA assessed associations between factors and self-reported PA; none assessed associations between factors and device-measured (e.g., accelerometers) PA. The 11 studies of eating behaviors assessed a diverse range of eating behaviors, including daily fruit and vegetable intake, red meat intake, fast food and sugary drink consumption, and overall diet quality or adherence to dietary guidelines, of which alcohol consumption was a component.

The number of studies that assessed each level of influence and the combinations of the levels for PA and eating behaviors are shown in Table 2. Of the 17 PA studies, 12 (71%) assessed only one level of influence, four (24%) assessed two levels of influence, and one (6%) assessed three levels of influence; thus, five studies examined two or more levels of influence simultaneously. Of the 11 eating behaviors studies, eight (73%) assessed only one level of influence, two (18%) assessed two levels of influence, and one (9%) assessed three levels of influence; thus, three studies examined two or more levels of influence simultaneously. None of the PA or eating behavior studies assessed all four levels of influence simultaneously.

### 3.2. Risk of Bias.

The quality ratings for individual studies are presented in Table 1. The two randomized controlled studies both had scores of 6 of 11 [21, 22], and the 21 observational studies had scores ranging from 13 of 19 [23] to 18 of 20 [24]. Most studies clearly stated their research aims and defined the target population, used appropriate measures to assess variables and adequately described results. However, only two studies reported a sample size calculation and justification [25, 26], and three studies had samples of fewer than 50 patients [23, 27, 28]. In addition, only two studies reported information about nonresponders [24, 29], and no studies reported measures undertaken to address nonresponders. For each study, we divided the number of criteria met by the total number of criteria included on the CASP checklist or AXIS tool to obtain a proportion describing the quality of the study. The average proportion was 0.77. Twelve studies (52%) had a proportion of at least 0.80 [24–26, 29–37]. Of these 12 studies, 11 investigated PA either solely [24–26, 30–32, 35, 37] or in addition to eating behaviors [29, 33, 34], nine focused on individual-level factors only [24, 26, 29–31, 34–37], and only three [25, 32, 33] assessed factors at other level(s) of influence beyond those at the individual level. However, due to the small number of studies that investigated the same factors within a certain level of influence, we could not draw firm conclusions from these higher-quality studies.

### 3.3. Multiple Levels of Influence on PA.

Of the 17 studies that investigated PA, 16 (94%) assessed individual-level factors, two (12%) assessed family/friend-level factors, one (6%) assessed provider/team-level factors, and four (24%) assessed organization/local community/policy environment-level factors. The multiple levels of influence and the corresponding factors that were examined for PA are shown in Table 3.

#### 3.3.1. Individual Level Factors.

At the individual level, demographic characteristics and cancer- and treatment-related factors were the most examined factors, and results were largely consistent across studies. Overall, these studies found no significant associations between PA and income level, education level, marital status, cancer stage, or cancer treatment type. Only one study found that education level was positively associated with PA among Black cancer survivors [30]. Studies assessing associations between PA and age or employment status yielded mixed results. For age, two studies reported a negative association with PA [37, 38], whereas three reported a positive association [31, 39, 40], and four reported no association [24, 26, 29, 32]. For employment status, one study reported a negative association [40], whereas two reported a positive association [25, 30], and three reported no association [28, 29, 37]. Regarding health status, three studies showed that comorbidity was negatively associated with PA [30, 32, 37]. Also, four studies showed inverse associations between anthropometrics (e.g., BMI) and PA [24, 26, 30, 37], and two studies showed positive associations between QoL and PA [25, 30]. However, the direction of the effect may have been from PA to each health status characteristic; that is, survivors who were not physically active were more likely to be overweight or obese, and those who were physically active were more likely to have higher QoL, particularly given that all but one study were cross-sectional studies. Eight studies investigated psychological factors, most of which were derived from social-cognitive theories [41, 42], such as self-efficacy, planning, and perceived benefits of and barriers to PA. Overall, self-efficacy, planning, and benefits were positively associated with PA [21, 25, 33], and perceived barriers were negatively associated with PA [28, 32]. Only one study examined life stress, which was not significantly associated with PA in Black breast cancer survivors [34].

#### 3.3.2. Factors Beyond the Individual Level of Influence.

Two studies investigated the family/social support level of influence. However, one of these studies (*N* = 62) found that friend support was positively associated with PA [25], whereas the other study (*N* = 246) did not find that family or friend support was associated with PA [33]. Only one study investigated the provider/team level of influence and showed that advice from health professionals was not significantly associated with PA in Black breast cancer survivors [38]. Finally, four studies assessed the organization/local community/policy environment level of influence, but their results were not conclusive. These studies showed that better access to exercise facilities (among Black breast cancer survivors) [33], a lower proportion of renters in the neighborhood (among Black breast cancer survivors) [32], and a higher degree of acculturation (among Asian American breast cancer survivors) [43] were significantly associated with higher PA, whereas perceived neighborhood safety [33], neighborhood characteristics (e.g., household income, poverty rate, and proportion of minority residents) [32], and environmental support (in a diverse group of breast cancer survivors) [28] were not.

### 3.4. Multiple Levels of Influence on Eating Behaviors.

The multiple levels of influence and their corresponding factors for eating behaviors are shown in Table 4. Eleven studies investigated a diverse range of eating behaviors, which included individual components of the diet, such as fruits and vegetables, fast food, sugary drinks, red meat, and fat, and overall eating quality or adherence to dietary guidelines. No study investigated alcohol consumption as a sole outcome. Instead, alcohol consumption was assessed only as a component of dietary guidelines [40].

#### 3.4.1. Individual Level Factors.

All 11 studies assessed at least individual-level factors. Overall, studies apiece found no significant associations between eating behaviors and age [29, 38, 44, 45], education level [29, 36, 38, 44], and disease- and treatment-related factors such as cancer stage [38, 44], treatment type [38, 44], and time since diagnosis or treatment completion [29, 44, 45]. However, findings for associations between income level and eating behaviors differed among studies. Some studies reported that a higher income level was associated with less red meat intake [44] and more fruit and vegetable intake [29, 44], whereas others reported no significant associations between income level and fast food and sugary drink consumption [29], dietary guideline adherence [45], or fruit and vegetable intake [27], although the latter study included only 24 participants. In the four studies that investigated psychological factors (e.g., self-efficacy, beliefs, stage of change, taste preference, stress) [22, 33, 34, 36], only self-efficacy was found to be positively associated with overall diet quality [33, 36].

#### 3.4.2. Factors Beyond the Individual Level of Influence.

Only a few studies examined factors other than those at the individual level; two assessed family/friend-level factors [23, 33], one assessed provider/team-level factors [38], and two assessed organization/local community/policy environmental-level factors [27, 33]. Thus, we could not derive conclusions from these studies. At the family/social support level, one study found that family support but not friend support was positively associated with healthy eating [33]. At the provider level, one study showed that advice from health professionals was positively associated with healthy eating [38]. At the organizational level, one study found no association between eating behaviors and perceived neighborhood safety and perceived access to healthy eating [33], and another study found no association between eating behaviors and objective measures of access to healthy eating [27].

## 4. Discussion

We found that almost all studies investigated factors at the individual level (16 out of 17 studies for PA and all 11 studies for diet). These studies assessing individual-level factors mostly focused on nonmodifiable factors, such as demographic characteristics and cancer- or treatment-related factors. Overall, the studies’ findings suggest that demographic and cancer treatment-related factors (e.g., age, level of education, cancer stage, treatment type) are not associated with PA and eating behaviors. Thus, interventions to improve PA and eating behaviors would be applicable to racial/ethnic minority survivors regardless of their demographics and treatment. In contrast, within the same individual level, fewer studies investigated psychological factors (eight studies for PA and four studies for eating behaviors), most of which were derived from social-cognitive theories (e.g., self-efficacy, planning, benefits, barriers). Overall, these studies showed these factors to be positively associated with PA. Thus, existing social-cognitive theories could be applied to design interventions to help improve racial/ethnic minority survivors’ PA, and individual-level psychological factors should be included in a multilevel PA intervention.

However, because few studies assessed factors other than individual-level factors, it is difficult to conclude which level(s) of influence should be targeted beyond the individual level of influence. While these studies measured diverse factors across the family/social support (e.g., social support from family or friends, social network diversity), provider/team (e.g., advice from health professionals), and organization/local community/policy environment (e.g., perceived access to exercise/healthy foods and neighborhood safety, acculturation) levels, each factor was investigated in only one or two studies. This lack of studies investigating multiple levels of influence represents a shortcoming in the development of interventions aimed at increasing PA and healthy eating among underserved survivor populations because qualitative studies have shown that subjective norms [46], communication with providers [47], providers’ recommendation of exercise [48], and family support [49] are important facilitators of PA engagement and/or healthy eating in racial/ethnic minority cancer survivors.

The results of the few studies we identified, in combination with the findings from the qualitative studies, could provide a basis for developing multilevel interventions. Specifically, for PA, one feasible example is to integrate healthcare providers/professionals (e.g., oncologists, oncology nurses, and primary care providers) into survivorship care while applying interventions targeting individual-level factors. In our review, we found only one study that investigated the provider/team level of influence on PA, and it showed that receiving exercise advice from healthcare professionals does not lead to an increase in PA among Black breast cancer survivors [38]. However, this result does not necessarily contradict the findings of existing studies. An intervention study conducted among Korean breast cancer survivors showed that survivors who received only oncologists’ PA recommendations did not increase their PA, whereas those who received an exercise motivation package in addition to oncologists’ recommendations significantly increased their PA [50]. Similarly, another intervention study conducted among breast cancer survivors showed that survivors in the intervention group, who received healthcare providers’ exercise advice plus telephone counseling, had greater improvements in PA than did those in the control group, who received the same advice from health care providers plus contacts from the interventionist [51]. Thus, a multilevel PA intervention that targets both individual- and provider-level factors may be designed and tested among racial/ethnic minority survivors.

For eating behaviors, even fewer studies that focused on racial/ethnic minority survivors’ eating behaviors existed, and they investigated a diverse range of eating behaviors, including individual components of the diet, overall diet quality, and adherence to dietary guidelines. Thus, we could find less consensus regarding the factors associated with eating behaviors. This suggests an urgent need for high-quality studies that assess eating behaviors across multiple levels of influence and focus on overall eating quality or adherence to dietary guidelines (e.g., Diet History Questionnaire [52]) as a diet outcome and whose results can be compared with an existing norm or directly compared with findings in other groups (e.g., NHW survivors).

In fact, to date, few diet interventions have been developed specifically for racial/ethnic minority survivors [53–55]. However, in the current review, the finding that family support is significantly associated with healthy eating improvement might serve as a good starting point to investigate factors other than those at the individual level. For example, a study that investigated the feasibility, acceptability, and preliminary efficacy of a lifestyle intervention for Latina cancer survivors and caregivers showed that this dyadic lifestyle intervention is feasible (63% consent rate) and efficacious to improve fruit and vegetable intake, sugar intake, and fiber intake among survivors [55]. The study’s results also showed that the intervention improved sugar intake and vegetable intake among caregivers. Such a dyadic-based approach that addresses both individual- and family-level influence could be valuable, especially for Hispanic cancer survivors who priotize familismo, which emcompasses commitment, loyalty, and dedication to the family [56].

This review also highlights a lack of studies among certain racial minority survivors. Specifically, only one study included Asian (Chinese) American survivors. In fact, to our knowledge, only one pilot lifestyle intervention specifically targeting Asian American (Chinese) survivors has been investigated [57]. However, Asian immigrants are the fastest-growing immigrant population in the United States [58], and the number of Asian American cancer survivors is expected to increase exponentially [2]. Furthermore, Asian Americans comprise diverse ethnic groups, and other Asian ethnic groups, such as Indian, Vietnamese, and Filipino groups, make up a large proportion of the Asian American population [58]. Yet much is still unknown regarding barriers to and facilitators of PA and eating behaviors among Asian American survivors. Thus, studies that investigate these lifestyle behaviors of Asian American survivors and assess interventions that target multiple levels of influence are urgently needed.

Consistent with other cancer survivorship studies, most of the studies included in this review focused on breast cancer survivors; there was a substantial lack of studies that included racial/ethnic minority survivors diagnosed with other types of cancer or that included male survivors. Although breast cancer is the most common cancer among racial/ethnic minority women [59], other types of cancer disproportionately affect minority men and women and require special attention. For example, compared with NHW individuals, Hispanic and Asian American populations have an approximately two-fold higher incidence of certain cancers, including liver and stomach cancers [60, 61]. Also, compared with NHW men, Black men have about 1.7 times the risk of developing prostate cancer and about 2.1 times the risk of dying of prostate cancer [62]. This dearth of studies may partly explain why very few culturally adapted lifestyle interventions are designed for racial/ethnic minority and nonbreast cancer survivors (see [63, 64]). Thus, much research involving survivors of cancers other than breast cancer is warranted.

Finally, none of the studies in this review included the use of accelerometers, such as ActiGraphs, to assess PA. In several studies, accelerometers were used among subsamples [21, 39], but the purpose of those studies was to compare accelerometer wear data with self-reported PA. However, analyzing ActiGraph data requires many resources and much time. Alternatives may include commercially available accelerometers or low- or no-cost smartphone applications. The latter is a feasible option because most members of racial/ethnic minority groups (e.g., 83% of Black people and 85% of Hispanic people) in the United States report owning a smartphone [65].

### 4.1. Study Limitations.

This systematic review had several limitations. First, this study could not be preregistered with the Systematic Review Protocol Registries because the database search was initiated before the registration. Second, there may be a risk of bias in the review procedures because the data coding was conducted by only the lead author, and thus, inter-rater reliability could not be calculated. Third, because the review included only published papers, its findings may reflect a publication bias. Fourth, the review did not include studies conducted outside the United States; however, because the United States has different social norms, environmental characteristics, and healthcare systems compared with other countries, focusing on studies conducted in the United States helped reduce heterogeneity. Fifth, because our aim was to evaluate associations between factors and lifestyle behaviors, this review included only quantitative studies. A future review of qualitative studies may provide a more contextual understanding of how different levels of influence and their factors affect PA and/or eating behaviors. Finally, although we used thorough search strategies, our use of four databases for the literature search may have been insufficient to capture all relevant studies.

## 5. Conclusions

In designing a multilevel intervention for PA or diet for racial/ethnic minority survivors, at the individual level, psychological factors from social-cognitive theories could be applied. The existing few systematic reviews investigating PA or diet among minority cancer survivors that have been published focused on Black patients [53, 66]. By including multiple minority groups and focusing on multiple levels of factors, the current systematic review, which was explicitly based on the socio-ecological model to code levels of influence, clearly showed that studies investigating multiple levels of influence beyond the individual level to improve minority cancer survivors’ PA and diet, particularly for Asian American survivors, male survivors, and survivors coping with cancers other than breast cancer are urgently needed. For eating behaviors, high-quality studies assessing overall eating quality or adherence to dietary guidelines are urgently needed.

## 6. Future Directions

We acknowledge the potential challenges in conducting studies that assess multiple levels of influence, particularly among racial/ethnic minority survivors, including slow recruitment and enrollment and demands for resources and time. In fact, some of the studies included in this systematic review had very small samples and thus were probably underpowered [23, 27, 28]. One potential strategy is to conduct multi-methods studies, such as those in which researchers first analyze publicly available large-scale datasets that include large numbers of racial/ethnic minority survivors and then conduct small-scale qualitative inquiries. Analyzing large datasets may provide empirical evidence about the existence and direction of interactions between different levels of influence, which could then be used to refine the social-ecological framework for influencing racial/ethnic minority survivors’ lifestyle behaviors. Qualitative inquiries will help us gain a more in-depth understanding of multiple factors, including cultural and contextual factors, and their interplay in influencing behavior. These efforts will eventually contribute to the successful development of multilevel, culturally sensitive interventions for minority cancer survivors.

## Supplementary Material

Suppl 1

## Figures and Tables

**Table 1:** Summary of the 23 studies included in this review.

First author, year	Sample size	Race/ethnicity	Cancer type	Level of influence examined	Behavior assessed	Study design	Quality rating

Beebe-Dimmer, 2020 [30]	1500	Black	Multiple	Individual	PA	Cross-sectional	17/20 (AXIS)
Crookes, 2016 [23]^a^	34	Hispanic	Breast	Family/social support	Diet	Cross-sectional	13/19 (AXIS)
Feathers, 2015 [27]^a^	24	Hispanic	Breast	Individual; organization/local community/policy environment	Diet	Longitudinal	15/19 (AXIS)
Ford, 2020 [31]	66	Black	Breast	Individual	PA	Cross-sectional	16/20 (AXIS)
Glenn, 2018 [29]	156	Multiple (24% Hispanic; 15% Black; 29% Asian)	Multiple	Individual	PA; diet	Cross-sectional	17/20 (AXIS)
Hair, 2014 [24]	830	Black	Breast	Individual	PA	Longitudinal	18/20 (AXIS)
Haymer, 2020 [40]	236	Multiple (62% Hispanic; 16% Black)	Prostate	Individual	PA; diet	Cross-sectional	15/20 (AXIS)
Jarvandi, 2021 [38]	228	Black	Breast	Individual; provider/team	PA; diet	Longitudinal	15/20 (AXIS)
Jones, 2016 [32]^b^	275	Black	Breast	Individual; organization/local community/policy environment	PA	Cross-sectional	16/20 (AXIS)
Kwarten, 2020 [33]^c^	246	Black	Breast	Individual; family/social support; organization/local community/policy environment	PA; diet	Longitudinal	16/20 (AXIS)
Kwarten, 2021 [34]^c^	246	Black	Breast	Individual	PA; diet	Longitudinal	16/20 (AXIS)
Le, 2019 [43]	195	Asian (Chinese American)	Breast	Organization/local community/policy environment	PA	Cross-sectional	15/20 (AXIS)
Mama, 2017 [21]^d^	89	Hispanic	Breast	Individual	PA	Longitudinal	6/11 (CASP)
Ortiz, 2018 [39]^d^	89	Hispanic	Breast	Individual	PA	Cross-sectional	14/20 (AXIS)
Paxton, 2019 [35]^b^	267	Black	Breast	Individual	PA	Cross-sectional	16/20 (AXIS)
Ramirez, 2016 [44]^e^	240	Black	Breast	Individual	Diet	Cross-sectional	14/20 (AXIS)
Rossi, 2017 [25]	62	Multiple (32% Black; 30% Hispanic)	Endometrial	Individual; family/social support	PA	Cross-sectional	17/20 (AXIS)
Smith, 2018 [26]^e^	193	Black	Breast	Individual	PA	Cross-sectional	16/20 (AXIS)
Shi, 2018 [22]^a^	70	Hispanic	Breast	Individual	Diet	Longitudinal	6/11 (CASP)
Spector, 2013 [28]	31	Multiple (65% Black; 35% Hispanic)	Breast	Individual; organization/local community/policy environment	PA	Cross-sectional	15/19 (AXIS)
Springfield, 2019 [45]^c^	210	Black	Breast	Individual	Diet	Cross-sectional	14/20 (AXIS)
Springfield, 2019 [36]^c^	210	Black	Breast	Individual	Diet	Cross-sectional	16/20 (AXIS)
Swen, 2017 [37]^b^	267	Black	Breast	Individual	PA	Cross-sectional	17/20 (AXIS)

PA, physical activity; AXIS, appraisal tool for cross-sectional studies; CASP, critical appraisal skills program.

a,b,c,d,e,f These studies used the same patient populations.

**Table 2:** Numbers and combinations of studies assessing one or more levels of influence.

Level of investigation	No. of studies	
	Physical activity (*n* = 17)	Eating behaviors (*n* = 11)

*1 level*		
Individual	11	7
Family/social support	0	1
Provider/team	0	0
Organization/local community/policy environment	1	0

*2 levels*		
Individual & family/social support	1	0
Individual & provider/team	1	1
Individual & organization/local community/policy environment	2	1
Family/social support & provider/team	0	0
Family/social support & organization/local community/policy environment	0	0
Provider/team & organization/local community/policy environment	0	0

*3 levels*		
Individual & family/social support & provider/team	0	0
Individual & family/social support & organization/local community/policy environment	1	1
Family/social support & provider/team & organization/local community/policy environment	0	0

*4 levels*		
Individual & family/social support & provider/team & organization/local community/policy environment	0	0

**Table 3:** Summary of 17 studies that assessed associations between physical activity and factors at different levels of influence.

Level (domain)	Factors	No. of studies examining the factor	Results		
			− (negative association)	+ (positive association)	≠ (null association)

Individual (demographics)	Age/age at dx	9	(37, 38)	(31, 39, 40)	(24, 26, 29, 30, 32)
	Sex	2		(30)^a^	(29)
	Education level	8		(30)	(24, 26, 28, 29, 32, 38, 39)
	Income level	4		(32)	(24, 26, 29)
	Employment status	6	(40)	(25, 30)	(28, 29, 37)
	Marital status	3			(24, 26, 29, 32)

Individual (cancer tx)	Site of cancer	1	(30)^b^	(30)^c^	
	Stage of cancer	6			(24, 26, 30, 32, 37, 38)
	Tx type	5	(30)	(26)	(24, 37, 38)
	Time since dx/tx completion	2		(37)	(29)

Individual (mental, physical, and overall health)	Self-reported health	5	(30)^d^	(25, 30)^e^, (35)	(38)
	Anthropometrics (e.g., BMI/waist circumference)	7	(24, 26, 30, 37)		(25, 38, 39)
	Comorbidity/physical fitness	7	(30, 32, 37)	(39)	(24, 26, 38)

Individual (psychological factor)	Self-efficacy	5		(21, 25, 33)	(28, 29)
	Planning/self-regulation	2		(35)	(25)
	Perceived barriers	2	(28)^f^, (32)		(28)^g^
	Benefits/outcome expectancy	2		(28)^f^	(25), (28)^g^
	Life stress	1			(34)

Family/social support	Family/friend support	2		(33)	(25)

Provider/team	Advice from health professionals	1			(38)

Organization/local community/policy environment	Neighborhood % renters	4	(32)		
	Neighborhood safety				(33)
	Neighborhood income, poverty rate, minority rate				(32)
	Access to exercise			(33)	
	Perceived environmental support				(28)
	Acculturation			(43)	

dx, diagnosis; tx, treatment

a being male

b lung cancer

c prostate cancer

d depressive and anxiety symptoms

e health-related quality of life

f Hispanic

g Black.

**Table 4:** Summary of 11 studies that assessed associations between eating behaviors and factors at different levels of influence.

Level (domain)	Factors	No. of studies examining the factors	Results		
			− (negative association)	+ (positive association)	≠ (null association)

Individual (demographics)	Age	4			(29, 38, 44, 45)
	Sex	1			(29)
	Education level	5		(45)	(29, 36, 38, 44)
	Income level	4	(44)^a^	(29, 44)^b^	(29)^c^ (27, 45)
	Employment status (employed)	3		(29)^d^	(29)^e^, (27, 44)
	Health insurance	1			(45)
	Marital status	4	(40)		(29, 44, 45)

Individual (cancer tx)	Stage of cancer/recurrence	2			(38, 44)
	Tx type	2			(38, 44)
	Time since dx/tx completion	3			(29, 44, 45)

Individual (mental, physical, and overall health)	Anthropometries (e.g., BMI/waist circumference)	4	(36)		(38, 44, 45)
	Comorbidity/physical fitness	2	(44)^f^		(38), (44)^g^
	HRQOL/general health	3		(40)	(29, 38)

Individual (psychological factor)	Self-efficacy	3		(33, 36)	(22)
	Beliefs	1			(22)
	Stage of change	1			(22)
	Taste/snack preference for F&V	1	(22)		
	Life stress	1			(34)

Family/social support	Social support	2		(33)^h^	(33)^i^
	Social network diversity and number of network members				(23)

Provider/team	Advice from health professionals	1		(38)	

Organization/local community/policy environment	Perceived access to healthy eating	2			(33)
	Food environment				(27)
	Perceived neighborhood safety				(33)

F&V, fruit and vegetable; tx, treatment; dx, diagnosis; BMI, body mass index; HRQOL, health-related quality of life.

a red meat intake

b F&V intake

c fast food and sugary drinks

d fast food

e F&V intake and sugary drinks

f physical functioning for red meat intake

g pain intensity for red meat intake

h family support

i friend support.

## Data Availability

The datasets generated and/or analyzed during the current study are available from the corresponding author upon reasonable request.
