# Supplementary material for: Multiple Levels of Influence on Lifestyle Behaviors among Cancer Survivors in Racial and Ethnic Minority Groups: A Systematic Review
Source: Eur J Cancer Care (Engl). Author manuscript; Available in PMC 2024 Dec 2. (PMC11611251; doi:10.1155/2023/8504968)
Supplement: Suppl 1 [file NIHMS2016038-supplement-Suppl_1.docx]

Table S1. Ovid MEDLINE search strategy

| # | Searches |
| --- | --- |
| 1 | exp *Survivors/ |
| 2 | (survivors adj3 (cancer* or neoplas* or tumor* or tumour* or leukaemia* or leukemia* or lymphoma* or oncolog*)).ti,ab,kf. |
| 3 | ((patients or survivors or group or groups or subgroups or population or populations or women or men or community or communities or neighborhood) adj20 cancer).ti. |
| 4 | (patients adj3 (cancer* or neoplas* or tumor* or tumour* or leukaemia* or leukemia* or lymphoma* or malignan* or oncolog* or carcinoma*)).ti. |
| 5 | ((cancer* or leukaemia* or leukemia* or lymphoma*) adj15 (ethnic* or racial* or minorit* or American or Americans)).ti. |
| 6 | 1 or 2 or 3 or 4 or 5 |
| 7 | exp *Neoplasms/ |
| 8 | (cancer* or neoplas* or tumo?r* or leuk?emia* or lymphoma* or malignan* or oncolog* or hematolo* or carcinoma*).ti,kf. |
| 9 | 7 or 8 |
| 10 | 6 and 9 |
| 11 | limit 10 to english language |
| 12 | (child or children or childhood or p?ediatric*).ti. |
| 13 | 11 not 12 |
| 14 | (cancer adj2 (awareness or prevention or screening or risk*)).ti. |
| 15 | *"Early Detection of Cancer"/ |
| 16 | 14 or 15 |
| 17 | cancer survivor*.ti. |
| 18 | 16 not 17 |
| 19 | 13 not 18 [remove some cancer screening articles] |
| 20 | exp African Americans/ |
| 21 | (african adj2 american*).ti,ab,kf. |
| 22 | exp asian americans/ |
| 23 | ((cambodian or korean or vietnamese or asian or chinese or filipino or hmong or japanese or indian) adj2 american*).ti,kf. |
| 24 | ((cambodian or korean or vietnamese or asian or chinese or filipino or hmong or japanese or indian) adj2 american*).ab. /freq=2 |
| 25 | exp hispanic americans/ |
| 26 | (Hispanic* or latino* or latina*).ti,kf. |
| 27 | (Hispanic* or latino* or latina*).ab. /freq=2 |
| 28 | (Puerto Rican* or Mexican* or Cuban* or Dominican*).ti,kf. |
| 29 | (Puerto Rican* or Mexican* or Cuban* or Dominican*).ab. /freq=2 |
| 30 | ((Central or South or spanish or latin) adj2 american*).ti,kf. |
| 31 | (minorit* or black or blacks).ti,kf. |
| 32 | (minorit* or black or blacks).ab. /freq=2 |
| 33 | exp Minority Groups/ |
| 34 | ((races or ethnic* or racial* or minorit*) and cancer).ti. |
| 35 | or/20-34 |
| 36 | 19 and 35 |
| 37 | exp *Health Behavior/ |
| 38 | (health* adj3 (lifestyle or "life style" or behavio?r*)).ti,ab,kf. |
| 39 | exp *Life Style/ |
| 40 | (lifestyle or "life style" or behavio?r*).ab. /freq=2 |
| 41 | exp *Exercise/ |
| 42 | (exercise or exercise* or "physical activit*").ti,kf. |
| 43 | exp *Alcohol Drinking/ |
| 44 | (Alcohol adj (Drinking or consumption)).ti,kf. |
| 45 | exp Healthy Diet/ |
| 46 | (healthy adj (diet or eating)).ti,kf. |
| 47 | exp Fruit/ or exp Vegetables/ |
| 48 | (fruit or vegetables).ti,kf. |
| 49 | (health adj5 (races or ethnic* or racial* or minorit*)).ti. |
| 50 | or/37-49 |
| 51 | 36 and 50 [minority cancer survivor health behaviour] |
| 52 | exp "Quality of Life"/ |
| 53 | "Quality of Life".ti,kf. |
| 54 | *"Activities of Daily Living"/ |
| 55 | physical functioning.ti,kf. |
| 56 | *Health Status/ |
| 57 | physical health.ti,kf. |
| 58 | exp *Emotions/ |
| 59 | emotional functioning.ti,kf. |
| 60 | *Mental Health/ |
| 61 | "mental health".ti,kf. |
| 62 | social* functioning.ti,kf. |
| 63 | Social Adjustment/ |
| 64 | *SPIRITUALITY/ |
| 65 | spirituality.ti,kf. |
| 66 | (adjustment* or well-being).ti,kf. |
| 67 | or/52-66 |
| 68 | 36 and 67 |
| 69 | 68 not 51 [minority cancer survivor quality of life] |

Table S2. EBSCO CINAHL search strategy

| # | Searches |
| --- | --- |
| S64 | S31 AND S63 |
| S63 | S48 OR S49 OR S50 OR S51 OR S52 OR S53 OR S54 OR S55 OR S56 OR S57 OR S58 OR S59 OR S60 OR S61 OR S62 |
| S62 | TI adjustment* or well-being |
| S61 | TI SPIRITUALITY |
| S60 | (MM "Spirituality") |
| S59 | (MM "Social Adjustment") |
| S58 | TI social* N3 functioning |
| S57 | TI "mental health" |
| S56 | (MM "Mental Health") |
| S55 | TI "emotional functioning" |
| S54 | (MM "Emotions+") |
| S53 | TI "physical health" |
| S52 | (MM "Health Status") |
| S51 | TI "physical functioning" |
| S50 | (MM "Activities of Daily Living") |
| S49 | TI "Quality of Life" |
| S48 | (MM "Quality of Life+") |
| S47 | S31 AND S46 |
| S46 | S32 OR S33 OR S34 OR S35 OR S36 OR S37 OR S38 OR S39 OR S40 OR S41 OR S42 OR S43 OR S44 OR S45 |
| S45 | TI (health N5 (races or ethnic* or racial* or minorit*)) |
| S44 | TI fruit or vegetables |
| S43 | (MM "Vegetables") |
| S42 | (MH "Fruit+") |
| S41 | TI healthy N2 (diet or eating) |
| S40 | (MM "Diet") |
| S39 | TI Alcohol N2 (Drinking or consumption) |
| S38 | (MH "Alcohol Drinking+") |
| S37 | TI exercise or exercise* or "physical activit*" |
| S36 | (MM "Exercise+") |
| S35 | (MM "Physical Activity") |
| S34 | (MM "Life Style") |
| S33 | TI health* N3 (lifestyle or "life style" or behavio?r*) |
| S32 | (MH "Health Behavior+") |
| S31 | S20 AND S30 |
| S30 | S21 OR S22 OR S23 OR S24 OR S25 OR S26 OR S27 OR S28 OR S29 |
| S29 | TI ( minorit* or black or blacks ) |
| S28 | (MH "Minority Groups") |
| S27 | TI ( ((Central or South or spanish or latin) N2 american*) ) |
| S26 | TI ( Hispanic* or latino* or latina* or Puerto Rican* or Mexican* or Cuban* or Dominican* ) |
| S25 | (MH "Hispanics") |
| S24 | TI ( (cambodian or korean or vietnamese or asian or chinese or filipino or hmong or japanese or indian) N2 american*) |
| S23 | (MH "Asians+") |
| S22 | TI african N2 american* OR AB african N2 american* |
| S21 | MH "Blacks" |
| S20 | S12 NOT S19 |
| S19 | S15 NOT S18 |
| S18 | S16 OR S17 |
| S17 | (MM "Cancer Survivors") |
| S16 | TI (survivors N3 (cancer* or neoplas* or tumor* or tumour* or leukaemia* or leukemia* or lymphoma* or oncolog*)) |
| S15 | S13 OR S14 |
| S14 | TI (cancer N2 (awareness or prevention or screening or risk or risks)) |
| S13 | (MM "Cancer Screening") |
| S12 | S10 NOT S11 |
| S11 | TI child or children or childhood or pediatric* or paediatric* |
| S10 | S6 AND S9 |
| S9 | S7 OR S8 |
| S8 | ( cancer* or neoplas* or tumo?r* or leuk?emia* or lymphoma* or malignan* or oncolog* or hematolo* or carcinoma* ) |
| S7 | (MM "Neoplasms") |
| S6 | S1 OR S2 OR S3 OR S4 OR S5 |
| S5 | TI ((cancer* or leukaemia* or leukemia* or lymphoma*) N15 (ethnic* or racial* or minorit* or American or Americans)) |
| S4 | TI (patients N3 (cancer* or neoplas* or tumor* or tumour* or leukaemia* or leukemia* or lymphoma* or malignan* or oncolog* or carcinoma*)) |
| S3 | TI ((patients or survivors or group or groups or subgroups or population or populations or women or men or community or communities or neighborhood) N15 cancer) |
| S2 | TI ( (survivors N3 (cancer* or neoplas* or tumor* or tumour* or leukaemia* or leukemia* or lymphoma* or oncolog*)) ) OR AB ( (survivors N3 (cancer* or neoplas* or tumor* or tumour* or leukaemia* or leukemia* or lymphoma* or oncolog*)) ) |
| S1 | (MH "Cancer Survivors") |

Table S3. Ovid PsycInfo search strategy

| # | Searches |
| --- | --- |
| 1 | exp Survivors/ |
| 2 | (survivor* adj3 (cancer* or neoplas* or tumor* or tumour* or leukaemia* or leukemia* or lymphoma* or oncolog*)).ti,ab,hw. |
| 3 | ((patients or survivors or group or groups or subgroups or population or populations or women or men or community or communities or neighborhood) adj20 cancer).ti. |
| 4 | exp patients/ |
| 5 | ((cancer* or leukaemia* or leukemia* or lymphoma*) adj15 (ethnic* or racial* or minorit* or American or Americans)).ti. |
| 6 | (patients adj3 (cancer* or neoplas* or tumor* or tumour* or leukaemia* or leukemia* or lymphoma* or malignan* or oncolog* or carcinoma*)).ti,hw. |
| 7 | or/1-6 |
| 8 | exp Neoplasms/ |
| 9 | (cancer* or neoplas* or tumo?r* or leuk?emia* or lymphoma* or malignan* or oncolog* or carcinoma*).ti,hw. |
| 10 | 8 or 9 |
| 11 | 7 and 10 |
| 12 | limit 11 to english language |
| 13 | (child or children or childhood or p?ediatric*).ti. |
| 14 | 12 not 13 |
| 15 | (cancer adj2 (awareness or prevention or screening or risk*)).ti. |
| 16 | exp *Cancer Screening/ |
| 17 | 15 or 16 |
| 18 | cancer survivor*.ti. |
| 19 | 17 not 18 |
| 20 | 14 not 19 |
| 21 | blacks/ |
| 22 | african cultural groups/ |
| 23 | (african adj2 american*).ti,ab,hw. |
| 24 | exp asians/ |
| 25 | ((cambodian or korean or vietnamese or asian or chinese or filipino or hmong or japanese or indian) adj2 american*).ti,ab,hw. |
| 26 | exp "latinos/latinas"/ |
| 27 | (Hispanic* or latino* or latina*).ti,ab,hw. |
| 28 | (Puerto Rican* or Mexican* or Cuban* or Dominican*).ti,ab,hw. |
| 29 | ((Central or South or spanish or latin) adj2 american*).ti,ab,hw. |
| 30 | exp Minority Groups/ |
| 31 | (minorit* or black or blacks).ti,hw. |
| 32 | (minorit* or black or blacks).ab. /freq=2 |
| 33 | ((races or ethnic* or racial* or minorit*) and cancer).ti. |
| 34 | alaska natives/ or american indians/ |
| 35 | or/21-34 |
| 36 | 20 and 35 |
| 37 | exp Health Behavior/ |
| 38 | (health* adj3 (lifestyle or "life style" or behavio?r*)).ti,hw. |
| 39 | (lifestyle or "life style" or behavio?r*).ab. /freq=2 |
| 40 | exp Lifestyle/ |
| 41 | (exercise or exercise* or "physical activit*").ti,hw. |
| 42 | exp physical activity/ |
| 43 | exp alcohol drinking patterns/ |
| 44 | exp alcoholism/ |
| 45 | (Alcohol adj (Drinking or consumption)).ti,hw. |
| 46 | exp Diets/ |
| 47 | exp Eating Behavior/ |
| 48 | (healthy adj (diet or eating)).ti,hw. |
| 49 | Fruit/ |
| 50 | (fruit or vegetables).ti,hw. |
| 51 | (health adj5 (race or race* or ethnic* or racial* or minorit*)).ti,hw. |
| 52 | or/37-50 |
| 53 | 36 and 52 [minority cancer survivor health behaviour] |
| 54 | exp "Quality of Life"/ |
| 55 | "Quality of Life".ti,hw. |
| 56 | exp Physical Health/ |
| 57 | exp "Activities of Daily Living"/ |
| 58 | physical functioning.ti,hw. |
| 59 | exp *EMOTIONS/ |
| 60 | emotional functioning.ti,hw. |
| 61 | exp Mental Health/ |
| 62 | "mental health".ti,hw. |
| 63 | exp adjustment/ |
| 64 | social* functioning.ti,hw. |
| 65 | exp well being/ |
| 66 | (adjustment or well-being).ti,hw. |
| 67 | exp SPIRITUALITY/ |
| 68 | spirituality.ti,hw. |
| 69 | or/54-68 |
| 70 | 36 and 69 [minority cancer survivor quality of life] |

Table S4. NLM PubMed

| # | Searches |
| --- | --- |
| #19 | #15 AND #18 |
| #18 | Search "Quality of Life"[tw] OR "Activities of Daily Living"[TW] OR "physical functioning"[TW] OR "Health Status"[TW] OR "physical health"[TW] OR Emotions[TW] OR Emotion[TW] OR emotional[ti] OR "Mental Health"[TW] OR social[TI] OR "Social Adjustment"[TW] OR spirituality[TW] OR adjustment[TI] OR adjustments[TI] OR well-being[TI] Sort by: PublicationDate |
| #17 | #15 AND #16 |
| #16 | Search Behavior[TW] OR Behaviour[TW] OR lifestyle[TW] OR "life style"[TW] OR exercise[TI] OR exercises[TI] OR "physical activity"[TI] OR "physical activities"[TI] OR Alcohol[TW] OR diet[TW] OR eating[TW] OR fruit[TW] OR vegetables[TW] OR Health[ti] Sort by: PublicationDate |
| #15 | #13 NOT #14 |
| #14 | Search child[TI] OR children[TI] OR childhood[TI] OR pediatric[TI] OR paediatric[TI] Sort by: PublicationDate |
| #13 | #11 NOT #12 |
| #12 | Search "cancer screening"[ti] Sort by: PublicationDate |
| #11 | #10 AND #7 |
| #10 | #8 OR #9 |
| #9 | Search (african[TW] OR cambodian[TW] OR korean[TW] OR vietnamese[TW] OR asian[TW] OR chinese[TW] OR filipino[TW] OR hmong[TW] OR japanese[TW] OR indian[TW] OR Central[TW] OR South[TW] OR spanish[TW] OR latin[TW]) AND (american[TW] OR americanS[TW]) Sort by: PublicationDate |
| #8 | Search minority[TW] OR minorities[TW] OR black[TW] OR blacks[TW] OR Hispanic[TW] OR Hispanics[TW] OR latino[TW] or latinos[TW] OR latina[TW] OR latinas[TW] OR "Puerto Rican"[TW] OR Mexican[TW] OR Mexicans[TW] OR Cuban[TW] OR Cubans[TW] OR Dominican[TW] OR Dominicans[TW] Sort by: PublicationDate |
| #7 | #6 AND #2 |
| #6 | #3 OR #4 OR #5 |
| #5 | Search (survivors[TI] OR Patients[TI]) AND (cancer[TI] OR neoplasm[TI] OR tumor[TI] OR tumour[TI] OR leukaemia[TI] OR leukemia[TI] OR lymphoma[TI] OR oncology[TI]) Sort by: PublicationDate |
| #4 | Search ((cancer[TI] OR leukaemia[TI] OR leukemia[TI] OR lymphoma[TI]) AND (ethnic[TI] OR ethnicity[TI] OR racial[TI] OR minority[TI] OR minorities[TI] OR American[TI] OR Americans[TI])) Sort by: PublicationDate |
| #3 | Search (group[ti] OR groups[ti] OR subgroups[ti] OR population[ti] OR populations[ti] OR women[ti] OR men[ti] OR community[ti] OR communities[ti] OR neighborhood[ti]) AND cancer[TI] Sort by: PublicationDate |
| #2 | Search pubstatusaheadofprint Sort by: PublicationDate |

Table S5. Summary of the 23 studies included in this review with digital object identifiers (DOIs)

| **First author, year** | **Sample size** | **Race/Ethnicity** | **Cancer type** | **Level of influence examined** | **Behavior assessed** | **Study design** | **Quality rating** |
| --- | --- | --- | --- | --- | --- | --- | --- |
| Beebe-Dimmer, 2020  [10.1002/cncr.32725](https://doi.org/10.1002/cncr.32725) | 1500 | Black | Multiple | Individual | PA | Cross-sectional | 17/20 (AXIS) |
| Crookes, 2016 [10.1007/s11764-015-0475-6](https://doi.org/10.1007/s11764-015-0475-6) | 34 | Hispanic | Breast | Family/social support | Diet | Cross-sectional | 13/19 (AXIS) |
| - Feathers, 2015 [10.1016/j.nutres.2015.04.007](https://doi.org/10.1016/j.nutres.2015.04.007) | 24 | Hispanic | Breast | Individual; Organization/local community/policy environment | Diet | Longitudinal | 15/19 (AXIS) |
| Ford, 2020  [10.1016/bs.acr.2020.01.005](https://doi.org/10.1016/bs.acr.2020.01.005) | 66 | Black | Breast | Individual | PA | Cross-sectional | 16/20 (AIXS) |
| Glenn, 2018  [10.1080/07347332.2018.1448031](https://doi.org/10.1080/07347332.2018.1448031) | 156 | Multiple (24% Hispanic; 15% Black; 29% Asian) | Multiple | Individual | PA; diet | Cross-sectional | 17/20 (AXIS) |
| - Hair, 2014 [10.1002/cncr.28630](https://doi.org/10.1002/cncr.28630) | 830 | Black | Breast | Individual | PA | Longitudinal | 18/20 (AXIS) |
| - Haymer, 2020 [10.1016/j.urolonc.2020.06.005](https://doi.org/10.1016/j.urolonc.2020.06.005) | 236 | Multiple (62% Hispanic; 16% Black) | Prostate | Individual | PA; diet | Cross-sectional | 15/20 (AXIS) |
| - Jarvandi, 2021 [10.1093/abm/kaaa020](https://doi.org/10.1093/abm/kaaa020) | 228 | Black | Breast | Individual; Provider/team | PA; diet | Longitudinal | 15/20 (AXIS) |
| - Jones, 2016 [10.1016/j.pmedr.2015.07.010](https://doi.org/10.1016/j.pmedr.2015.07.010) | 275 | Black | Breast | Individual; Organization/local community/policy environment | PA | Cross-sectional | 16/20 (AXIS) |
| - Kwarten, 2020 [10.1007/s10552-020-01315-y](https://doi.org/10.1007/s10552-020-01315-y) | 246 | Black | Breast | Individual; Family/social support; Organization/local community/policy environment | PA; diet | Longitudinal | 16/20 (AXIS) |
| - Kwarten, 2021 [10.1007/s11764-021-01054-2](https://doi.org/10.1007/s11764-021-01054-2) | 246 | Black | Breast | Individual | PA; diet | Longitudinal | 16/20 (AXIS) |
| - Le, 2019 [10.1007/s10903-018-0721-x](https://doi.org/10.1007/s10903-018-0721-x) | 195 | Asian (Chinese American) | Breast | Organization/local community/policy environment | PA | Cross-sectional | 15/20 (AXIS) |
| - Mama, 2017 [10.1002/pon.4026](https://doi.org/10.1002/pon.4026) | 89 | Hispanic | Breast | Individual | PA | Longitudinal | 6/11 (CASP) |
| - Ortiz, 2018 [10.1080/09593985.2018.1424978](https://doi.org/10.1080/09593985.2018.1424978) | 89 | Hispanic | Breast | Individual | PA | Cross-sectional | 14/20 (AXIS) |
| - Paxton, 2019 [10.1080/13557858.2017.1378805](https://doi.org/10.1080/13557858.2017.1378805) | 267 | Black | Breast | Individual | PA | Cross-sectional | 16/20 (AXIS) |
| - Ramirez, 2016 [10.21633/jgpha.6.2s06](https://doi.org/10.21633/jgpha.6.2s06) | 240 | Black | Breast | Individual | Diet | Cross-sectional | 14/20 (AXIS) |
| - Rossi, 2017 ( [10.1007/s00520-017-3622-y](https://doi.org/10.1007/s00520-017-3622-y) | 62 | Multiple (32% Black; 30% Hispanic) | Endometrial | Individual; Family/social support | PA | Cross-sectional | 17/20 (AXIS) |
| - Smith, 2018 [10.1080/13557858.2016.1256376](https://doi.org/10.1080/13557858.2016.1256376) | 193 | Black | Breast | Individual | PA | Cross-sectional | 16/20 (AXIS) |
| - Shi, 2018 [10.1002/pon.4799](https://doi.org/10.1002/pon.4799) | 70 | Hispanic | Breast | Individual | Diet | Longitudinal | 6/11 (CASP) |
| - Spector, 2013 [10.1188/13.ONF.472-480](https://doi.org/10.1188/13.onf.472-480) | 31 | Multiple (65% Black; 35% Hispanic) | Breast | Individual; Organization/local community/policy environment | PA | Cross-sectional | 15/19 (AXIS) |
| - Springfield, 2019 [10.1007/s11764-019-00748-y](https://doi.org/10.1007/s11764-019-00748-y) | 210 | Black | Breast | Individual | Diet | Cross-sectional | 14/20 (AXIS) |
| - Springfield, 2019 [10.1080/01635581.2018.1557217](https://doi.org/10.1080/01635581.2018.1557217) | 210 | Black | Breast | Individual | Diet | Cross-sectional | 16/20 (AXIS) |
| - Swen, 2017 [10.5888/pcd14.170128](https://doi.org/10.5888/pcd14.170128) | 267 | Black | Breast | Individual | PA | Cross-sectional | 17/20 (AXIS) |
